# Bark of *Passiflora edulis* Treatment Stimulates Antioxidant Capacity, and Reduces Dyslipidemia and Body Fat in *db*/*db* Mice

**DOI:** 10.3390/antiox7090120

**Published:** 2018-09-08

**Authors:** Marielle Fernanda Panelli, Damiana Tortolero Pierine, Sérgio Luiz Borges de Souza, Artur Júnio Togneri Ferron, Jéssica Leite Garcia, Klinsmann Carolo dos Santos, Matheus Antônio Filiol Belin, Giuseppina Pace Pereira Lima, Milena Galhardo Borguini, Igor Otávio Minatel, Antônio Carlos Cicogna, Fabiane Valentini Francisqueti, Camila Renata Corrêa

**Affiliations:** 1 Medical School, UNESP—São Paulo State University, Botucatu 18618-687, Brazil; mariellepanelli@gmail.com (M.F.P.); damiana.tp@gmail.com (D.T.P.); enfeborges@gmail.com (S.L.B.d.S.); artur.ferron@gmail.com (A.J.T.F.); jessleitegarcia@gmail.com (J.L.G.); klinsmanncarolo@gmail.com (K.C.d.S.); igorminatel@hotmail.com (I.O.M.); cicogna@fmb.unesp.br (A.C.C.); fabiane_vf@yahoo.com.br (F.V.F.); 2 Bioscience Institute, UNESP—São Paulo State University, Botucatu 18618-689, Brazil; matmem.belin@gmail.com (M.A.F.B.); gpplima@ibb.unesp.br (G.P.P.L.); mgborguini@hotmail.com (M.G.B.)

**Keywords:** *db*/*db* mice, obesity, *Passiflora edulis*, oxidative stress

## Abstract

Obesity is considered an important risk factor for several disorders, such as diabetes mellitus, systemic arterial hypertension, dyslipidemia, and atherosclerosis, which are associated with inflammation and oxidative stress as a trigger factor. *Passiflora edulis* contains important bioactive compounds, such as phenolics, carotenoids, vitamin C, and polyamines in pulp, leaves, seeds, and bark. **Aim**: To evaluate the effect of bark of *Passiflora edulis* (BPe) on body composition, and metabolic and oxidative stress parameters in genetically obese mice. **Methods**: Obese male *db*/*db* mice (*n* = 14 animals) received normal feeds and water ad libitum for 8 weeks. Then, animals were randomly divided to continue either receiving standard chow (obese, *n* = 7 (OB)) or feed with standard chow plus bark *Passiflora edulis* (BPe) (obese + BPe, *n* = 7 (OB + BPe)) for 8 more weeks, totaling 16 weeks. BPe was added to chow (7 g of BPe/kg of chow corresponding to 1.5 g/kg of body weight). The parameters evaluated in animals included food and caloric intake, body weight, body fat, plasma glucose, triglycerides, and total cholesterol. Malondialdehyde and antioxidant capacity were evaluated in plasma and organs. Groups were compared by Student *t*-test, with *p* < 0.05. **Results**: BPe reduced visceral and subcutaneous fat deposit and adiposity index, cholesterol and triglyceride levels, ameliorated the antioxidant capacity, and reduced malondialdehyde (MDA) levels. **Conclusion**: the bark of *Passiflora edulis* was effective in improving body composition, and metabolic and antioxidant parameters in obese mice.

## 1. Introduction

Epidemiological studies indicate that the high consumption of fruits and vegetables, whole grains, and fish have been associated with a lower risk of cardiovascular diseases and mortality, due to the bioactive compounds present in these foods [1,2,3]. However, in a new paradigm, their insufficient intake in the diet, as well the excessive consumption of energy, constitute an important risk factor for the development of diseases, mainly obesity [4].

According to the World Health Organization (WHO, (Geneva, Switzerland), obesity is defined as the excessive or abnormal accumulation of body fat which may cause health damage [5]. It is considered an important risk factor for several disorders, such as diabetes mellitus, systemic arterial hypertension, dyslipidemia, atherosclerosis, non-alcoholic fatty liver disease, heart and kidney failure, and some types of cancer [6,7,8]. Inflammation and oxidative stress in adipose tissue, due to adipocyte hypertrophy, are mechanisms suggested as cause of these disorders [9]. The excessive supply of foods increases the production of free radicals due the oxidation of these nutrients, which favors the inflammatory cells infiltration and oxidative stress [10]. Initially, this situation is concentrated in adipose tissue, but with a chronic period of obesity, oxidative stress and inflammation can also affect other organs and their function [11,12].

Oxidative stress is controlled by the endogenous antioxidant defense system, which includes antioxidant enzymes [13] and exogenous antioxidants from fruits and vegetables, such as vitamins C, flavonoids, vitamins E, and carotenoids ^14^. Studies have already shown health benefits from food antioxidants due to the action of endogenous antioxidants against free radicals [14,15,16]. Therefore, considering the complications caused by oxidative stress and inflammation in obese people, the introduction of natural antioxidants in the diet and their effect on health maintenance/recovery has become the focus of the scientific community. 

There are more than 500 *Passiflora* species known for their edible fruits, ornamental flowers, and pharmaceutical uses. They are extensively cultivated in tropical and subtropical regions of the world, especially in Central and South America [17,18]. *Passiflora edulis* has received attention in research due to the presence of important bioactive compounds, such as phenolics, carotenoids, vitamin C, and polyamines in pulp, leaves, seeds, and bark [19,20]. Bark represents more than a half of fruit (52%), but at the same time, it is the lesser used part. It is considered a functional food, since contains bioactive compound—fibers—able to reduce the risk of diabetes and cardiovascular disease (CVD) [21,22,23]. The literature has already reported that passionflower bark flour (*Passiflora edulis*) is rich in pectin, a soluble fiber, able to form a gel, preventing the absorption of cholesterol and glucose from the diet. As a positive effect, it reduces glucose intolerance in diabetic patients, and decreases serum cholesterol and triglyceride levels [24].

However, even with the benefits from pectin and antioxidant compounds in the bark of *Passifora edulis* [24,25,26], this fruit remains little explored in studies regarding obesity-related disorders. Bark of *Passifora edulis* (BPe) could be used as treatment against obesity, since bioactive compounds can reduce oxidative stress, delaying or preventing the manifestation of complications in target organs such as heart, liver, and kidney. Therefore, the aim of this study was to evaluate the effect of bark of *Passiflora edulis* (BPe) on body composition, and metabolic and oxidative stress parameters in genetically obese mice.

## 2. Materials and Methods

### 2.1. Animals and Experimental Protocol

In this study, *db*/*db* mice genetically mutated in the leptin receptor were used, for being a good rodent model for obesity and type 2 diabetes. All the animals were acquired from Universidade de São Paulo—USP, Brazil. Obese male *db*/*db* mice (*n* = 14 animals) received normal feed and water ad libitum (Presence for rats and mice, Presence Nutrição Animal, Brazil) for 8 weeks. Then, animals were randomly divided to continue receiving standard chow (obese, *n* = 7 (OB)) or standard chow plus bark *Passiflora edulis* (BPe) (obese + BPe, *n* = 7 (OB + BPe) for 8 more weeks, totaling 16 weeks. In order to confirm obesity, lean mice of the same lineage (*n* = 10) and same age, fed only standard diet, were used as the control group. For all animals, feed and water were offered ad libitum. Feed and water consumption were by measured daily, and body weight was measured weekly. Two animals per cage were kept in an environment with controlled temperature (24 ± 2 °C) and humidity (55 ± 5%) and light–dark cycle (12–12 h). The study protocol was approved by the Ethics Committee on Animal Experimentation of the Botucatu Medical School, Universidade Estadual Paulista—UNESP, (1104/2017) in São Paulo, Brazil, and followed the recommendations of the *Guide for the Care and Use of Experimental Animals* [27]. At the end of the experiment, the animals were euthanized, and the organs and blood were collected for analysis.

### 2.2. Preparation of Chow with Bark of Passiflora Edulis

*Passiflora edulis* fruits, from a rural producer in Presidente Prudente city, São Paulo, Brazil, in maturation stage, were submitted to selection and sanitization with deionized water. The fruit bark (exocarpium and mesocarpium) was washed, cut into small pieces, and oven-dried at 60 °C for 48 h. After this process, the dried barks were crushed in a mill type (Logen Scientific, Diadema São Paulo, Brazil). The commercial chow (Presence, Paulínea, São Paulo, Brazil) was ground, and then 7 g of *Passiflora edulis* bark/kg of chow was added (correspondent to 1.5 g/kg of body weight per day) [28]. Following this, the moisture was pelleted again for consumption.

### 2.3. Bark of Passiflora Edulis Antioxidants Characterization

#### 2.3.1. Carotenoids

Bark of *Passiflora edulis* (400 mg) was homogenized in 2.5 mL MeOH (High Performance liquid cromatograph (HPLC) grade) for 1 min using a vortex, and then ultrasonicated for 30 min at 25 °C. After centrifugation (10 min, 3800× *g*, Hettich Zentrifugen, Mikro220R, Tuttlingen, Germany), the supernatant was transferred to amber tubes. The residual pellet was submitted to one more process of extraction with 2.5 mL MeOH, and two more similar extractions with tetrahydrofuran (THF) (2.5 mL in each extraction). The supernatants (10 mL) were completely evaporated under N_2_. The dry organic solvent extract was resolubilized in 1 mL ethanol and centrifuged (5 min, 800*g* at 4 °C) and 20 μL of supernatant was injected into the HPLC/Diode array detector (DAD) system. The used method was described by Yeum et al. (1996) [29]: briefly, the system consisted of a reversed phase HPLC Thermo Scientific Ultimate 3000 (Thermo Scientific, San Jose, CA, USA), equipped with a diode array detector and C30 column for carotenoids (5 μm, 150 × 4.6 mm, YMC-Yamamura Chemical Research, Wilmington, NC, USA). HPLC mobile phase conditions consisted of methanol/methyl tert-butyl ether/water (85:12:3, *v/v/v*) with 1.5% ammonium acetate in water for solvent A and methanol/MTBE/water (8:90:2 *v/v/v*) with 1.0% ammonium acetate in water for solvent B. The solvent gradient was as follows: 90% A and 10% B (2 min), 85% A and 15% B (5 min), 75% A and 25% B (9 min), 60% A and 40% B (12 min), 17% A and 83% B (16 min), 5% A and 95% B (23 min), 5% A and 95% B (25 min), and 60% A and 40% B (28 min) at a flow rate of 0.4 mL min (10 °C). Carotenoids were quantified at 450 nm by determining peak areas under the curve in the HPLC calibrated against known amounts of standards. The results were adjusted using a standard curve for β-carotene, criptoxantin, and lutein (Sigma-Aldrich Co., St. Louis, MO, USA) of 99.98% purity. The recovery standard average was 97%. The results are expressed in ng/100 g, and correspond to the average of three consecutive injections per sample (*n* = 3). All analyses were performed in triplicate [29].

#### 2.3.2. Determination of 2, 2-Diphenyl-1-picrylhydrazyl radical (DPPH) Radical Scavenging Activity

The free radical scavenging hydrophilic capacity was performed. Three hundred microliters were added to 2 mL of DPPH solution (0.004% in ethanol). All samples were conducted in triplicate, and the readings were performed at 517 nm. Absorbance was converted to the percentage of antioxidant activity using the following equation: % Reduced DPPH = ((blank absorbance − sample absorbance)/blank absorbance) × 100. A calibration curve was prepared with Trolox (6-hydroxy-2,5,7,8-tetramethylchroman-2-carboxylic acid) and the results were expressed in µM equiv Trolox per 100 g dry weight (DW) [30].

#### 2.3.3. Determination of Total Phenol Content

Total phenolic content was determined using the Folin–Ciocalteu reagent Folin–Ciocalteu reagent was diluted 10 times with distilled water. The pepper extract solution (500 μL) was mixed with 1 mL diluted Folin–Ciocalteu reagent, 1 mL sodium bicarbonate solution (7.5%), and 1 mL distilled water. The mixture was incubated at room temperature for 15 min. The absorbance of the solution was determined at 730 nm using a spectrophotometer (Biowave, S2100, Cambridge, UK) and compared with gallic acid equivalents (GAE) calibration curve. The total phenolic content was expressed as mg gallic acid equivalents of 100 g of *Passiflora edulis* bark [31].

### 2.4. Nutritional Parameters

Nutritional profile was characterized by initial body weight (IBW), final body weight (FBW), total body fat (TF—sum of fat deposits: epididymal, retroperitoneal, visceral, and subcutaneous), adiposity index (AI—total body fat/final body weight × 100) and feed intake (g/day).

### 2.5. Metabolic Parameters

After 8 h fasting, serum levels of glucose, triglycerides, and cholesterol (kits from BioClin^®^, Belo Horizonte, MG, Brazil) were determined by an automatic enzymatic analyzer system (Chemistry Analyzer BS-200, Mindray Medical International Limited, Shenzhen, China).

### 2.6. Preparation of Tissues for Oxidative Stress Analysis

Increased reactive oxygen species (ROS) lead to oxidative stress, which is responsible for the oxidation of biomolecules and affecting antioxidant capacity. For this reason, we analyzed, in this study, both malondialdehyde levels (MDA), an important lipid peroxidation biomarker and antioxidant capacity in serum and tissues (adipose, hepatic, cardiac, and renal).

Tissues (100 mg) were homogenized in 1 mL of cold phosphate saline buffer (PBS), pH = 7.4, and centrifuged (800× *g*, 4 °C, 10 min). The supernatant was used in the following analyses. 

#### 2.6.1. Malondialdehyde (MDA)

Homogenate (100 μL) was used for MDA analysis. Briefly, 700 μL of 1% orthophosphoric acid and 200 μL of thiobarbituric acid (42 mM) were added to the sample. Then, the sample was boiled for 60 min in a water bath, and afterwards, it was immediately cooled on ice. Two hundred microliters was transferred to a 2 mL tube containing 200 μL sodium hydroxide/methanol (1:12 *v/v*). The sample was vortex-mixed for 10 s, and centrifuged for 3 min at 13,000× *g*. The supernatant (200 μL) was transferred to a 300 μL glass vial and 50 μL injected onto the column. The HPLC was a Shimadzu LC-10AD system (Kyoto, Japan) equipped with a C18 Luna column (5 μm, 150 × 4.60 mm, Phenomenex Inc., Torrance, CA, USA), a Shimadzu RF-535 fluorescence detector (excitation 525 nm, emission 551 nm), and 0.5 mL/min flow of phosphate buffer (KH2PO4 1mM, pH 6.8) [32]. MDA was quantified by determination of the peak area in the chromatograms relative to a standard curve of known concentrations.

#### 2.6.2. Antioxidant Capacity

The hydrophilic antioxidant capacity was determined fluorometrically, as described by Beretta et al. (2006) [33] using VICTOR X2 reader (Perkin Elmer-Boston, MA, USA). The antioxidant capacity was quantified by the comparison between the areas on the curve relative to the oxidation kinetics of the phosphatidylcholine (PC) suspension, which was used as a reference for biological matrix. 2,2′-Azobis (2-aminopropano)-dihydrochloride (AAPH) was used as the peroxyl radical initiator. The results represent the percentage of inhibition of 4,4-difluoro-5-(4-phenyl-1,3-butadienyl)-4-bora-3a,4a-diaza-s-indacene-3-undecanoic acid) (BODIPY, 581/591) in plasma relative to that occurring in the control sample of BODIPY 581/591 in PC liposome. All analyses were performed in triplicate, and the results represent the percent protection.

## 3. Statistical Analysis

Data were analyzed by Student *t*-test or Mann–Whitney, and the results are presented as means ± standard deviation (SD), or medians (interquartile range). Statistical analyses were performed using Sigma Stat for Windows Version 3.5. (Systat Software, Inc., San Jose, CA, USA), and a *p* value of 0.05 was considered as statistically significant.

## 4. Results

### 4.1. Bark of Passiflora Edulis Antioxidants Characterization

Table 1 presents the antioxidants characterization. It was possible to verify the presence of some carotenoids—β-carotene, lutein, cryptoxanthin, and also the amount of total phenolic. The table also presents the antioxidant capacity (DHPP).

### 4.2. Bark of Passiflora Edulis Reduced Visceral and Subcutaneous Fat Deposit and Adiposity Index

The treatment with BPe promoted a significant reduction of 34.5% in the visceral fat, 27.2% in subcutaneous and 16.1% in adiposity index when compared to the untreated group (Table 2). This positive effect happened even with a higher feed intake by the supplemented group.

### 4.3. Bark of Passiflora Edulis Reduced Cholesterol and Triglycerides Levels

**Figure 1:**
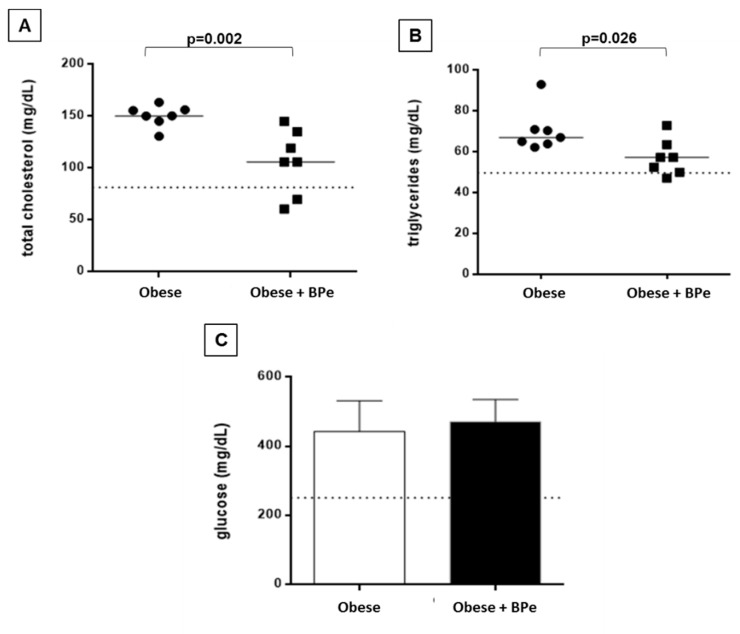
Plasma biochemical parameters for (**A**) total cholesterol; (**B**) triglycerides; (**C**) glucose. Data are expressed in mean ± standard deviation, or median. Dotted line represents mean value of control group. BPe—bark of *Passiflora edulis*.

The treatment with BPe was effective in reducing total cholesterol and triglyceride levels. No change in glucose level was observed (Figure 1).

### 4.4. Bark of Passiflora Edulis Ameliorates the Antioxidant Capacity and Reduces MDA Levels

**Figure 2:**
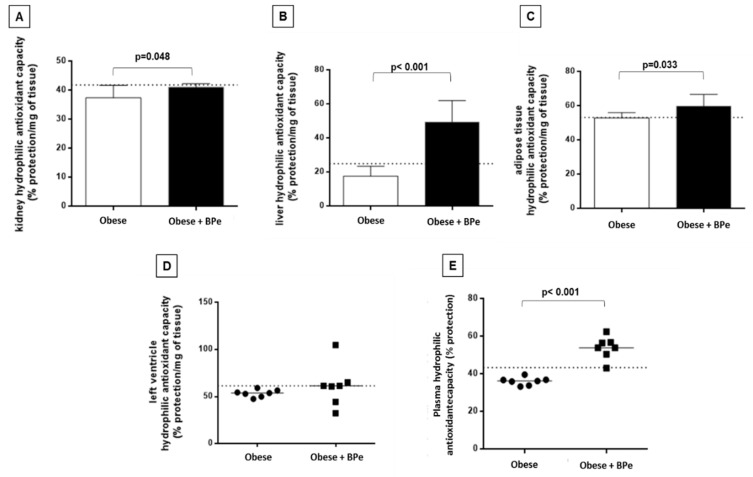
Antioxidant capacity in (**A**) kidney; (**B**) liver; (**C**) adipose tissue; (**D**) left ventricle; (**E**) plasma. Data are expressed in mean ± standard deviation, or median. Dotted line represents mean value of control group. BPe—bark of *Passiflora edulis*.

**Figure 3:**
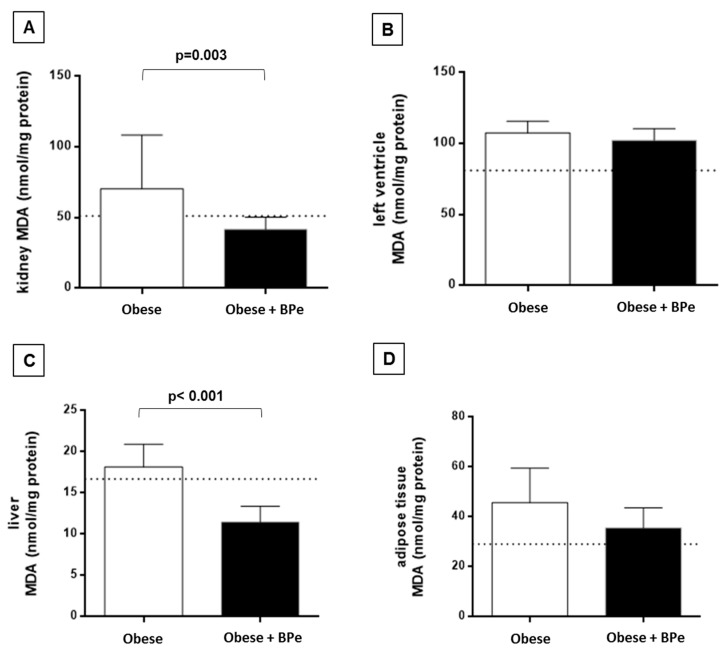
Malondialdehyde levels (MDA) in (**A**) kidney; (**B**) left ventricle; (**C**) liver; (**D**) adipose tissue. Data are expressed in mean ± standard deviation, or median. Dotted line represents means value of control group. BPe—bark of *Passiflora edulis*.

The treatment with bark of *Passiflora edulis* increased the antioxidant capacity in plasma, kidney, liver, and adipose tissue compared to the group without the compound (Figure 2). Regarding MDA levels, the bark was effective in reducing lipid oxidation in kidney and liver (Figure 3).

## 5. Discussion

Obesity is a serious health problem around the world. However, the most commonly used strategies for prevention or treatment of obesity (diet, exercise, drugs, and surgery) have some restrictions and side effects. Therefore, investigating new therapies for its prevention and treatment is very urgent [34]. In this study, we evaluated the effect of bark of *Passiflora edulis* on body composition, and metabolic and oxidative stress parameters in genetically obese mice. This animal model presents leptin receptor deficiency, and has served as a rodent model for obesity and type 2 diabetes for more than 40 years [35].

More important than the extent of body fat accumulation, the severity of obesity-related diseases is associated to body fat distribution. Visceral fat correlates significantly with glucose area after oral glucose tolerance test, with cholesterol and triglyceride levels, and increased risk for cardiac disease [36,37]. Regarding obesity parameters, the bark showed a positive effect in reducing visceral and subcutaneous adipose tissues and adiposity index. We did not find studies about BPe to compare the results, but Su (2016) [38] studying acetylshikonin, and Zhang (2012) [39] working with rhein, found similar results. Some research, regarding adipose tissue and bioactive compounds, showed that the body fat reduction is associated, in part, with an increase of the mitochondrial uncoupling protein-1 (UCP-1) in white adipose tissue, which is able to increase the metabolic energy heat [40,41].

Dyslipidemia and hyperglycemia are metabolic disorders associated with an increase of cardiovascular risk [42]. Regarding these parameters, the group treated with bark of *Passiflora edulis* presented a reduction in cholesterol and triglycerides levels, even in this animal model. Since *db*/*db* mice have the highest plasma triglyceride and cholesterol due to genetic mutation, the decrease of these parameters represents a relevant effect of BPe. One explanation for this positive effect is that *Passiflora edulis*, one of the various species of *Passiflora* genus plants [43], is rich in pectin, a soluble fiber with benefits to health [28,44,45]. Soluble fiber may increase short-chain fatty acid synthesis, thereby reducing endogenous cholesterol production, and a diet low in cholesterol and saturated fat can reduce blood levels of cholesterol and triacylglycerol [42,46].

Oxidative stress is a condition associated with development of many complications and comorbidities in obesity [16,47]. The bark of *Passiflora edulis* used in this study was effective in improving antioxidant defense in plasma, liver, kidney, and adipose tissue of *db*/*db* mice, in addition to a reduction of MDA in liver and kidneys. In this case, the increase of antioxidant defense and decrease in lipid peroxidation are relevant facts, since these conditions may avoid the manifestation of oxidative stress associated diseases, such as hepatic and renal failure. These findings can be associated with the bioactive compounds, as carotenoids and phenolic compounds present in the bark used in this study [28].

Carotenoids are pigments responsible for pigmentation in animals, plants, and microorganisms, but also with an important role in biological systems, especially as antioxidants [48]. The most important carotenoids include β-carotene, α-carotene, lycopene, lutein, zeaxanthin, and β-cryptoxanthin. Together with their metabolites, these substances are the most efficient physical and chemical scavengers of singlet oxygen and other reactive oxygen species (ROS) [49]. Considering that ROS are related to many disorders—cardiovascular disease, cancer, and neurodegenerative—the consumption of fruits and vegetables rich in carotenoids, such as *Passiflora edulis*, is important to reduce the risk for disorders.

Phenolic compounds are a class of compounds which contain an aromatic ring bearing one or more hydroxy substituent [50,51]. Fruits (grapes, apple, pear, cherries, and berries), vegetables, cereals, chocolate, and beverages (red wine, tea and coffee) are examples of dietary compounds containing polyphenols. Antioxidant activities from these substances includes suppression of ROS formation by the inhibition of enzymes involved in their production, scavenging of ROS, and upregulation or protection of antioxidant defenses [52]. Therefore, the daily intake of food rich in polyphenols may exhibit benefic effects to health.

## 6. Conclusions

In summary, this study presented benefits from bark of *Passiflora edulis* towards the health of genetic obese animals, reducing body fat and malondialdehyde, and improving dyslipidemia and antioxidant capacity. Hence, it is possible to conclude that the bark of *Passiflora edulis* is effective to improve body composition, and metabolic and antioxidant parameters in obese mice.

## Figures and Tables

**Table 1:** Bark of *Passiflora edulis* antioxidant characterization.

Parameters	Content/Activity
β-carotene	0.321 mg/100 mg DW
Lutein	57.53 mg/100 mg DW
Cryptoxanthin	2.23 mg/100 mg DW
Total phenolic	248.9 mg gallic acid equivalents/100 g DW
DHPP	1.1 µM equiv Trolox/100 g DW

DW—dry weight. DHPP: 2,2-Diphenyl-1-picrylhydrazyl radical.

**Table 2:** Nutritional status parameters.

Variables	Obese	Obese + BPe
**Feed intake (g/day)**	6.50 ± 0.15	10.8 ± 0.16 *
**IBW (g)**	17.6 ± 4.4	18.9 ± 4.4
**FBW (g)**	53.9 (0.6)	52.7 (18.5)
**RAT (g)**	1.87 (0.36)	1.43 (1.24)
**EAT (g)**	2.80 ± 0.33	2.85 ± 0.80
**VAT (g)**	1.80 ± 0.14	1.18 ± 0.51 *
**SAT (g)**	9.11 ± 1.19	6.58 ± 2.95 *
**TF (g)**	15.8 (2.0)	14.2 (7.9)
**AI (%)**	29.3 ± 2.5	24.66 ± 6.08 *

BPe—bark of *Passiflora edulis*; IBW—initial body weight; FBW—final body weight; RAT—retroperitoneal adipose tissue; EAT—epididymal adipose tissue; VAT—visceral adipose tissue; SAT—subcutaneous adipose tissue; TF—total fat; AI—adiposity index. Data are expressed in mean ± standard deviation, or median and interquartile range (FBW, RAT, TF, AI). * indicates *p* < 0.05.
